# First quantitative assessment of the effects of parasite control in dogs and cats in the UK

**DOI:** 10.1186/s13071-025-07137-8

**Published:** 2025-12-05

**Authors:** Alessio Giannelli, Alistair Antonopoulos, Hany M. Elsheikha, Ian Wright, Johannes Charlier

**Affiliations:** 1 Kreavet, Kruibeke, Belgium; 2 Faculty of Medicine and Health Sciences, School of Veterinary Medicine and Science, University of Nottingham, Loughborough, UK; 3 ESCCAP UK & Ireland, Hills Science Park, Malvern, UK; 4 Mount Veterinary Practice, Fleetwood, UK; 5 ESCCAP, Hills Science Park, Malvern, UK

**Keywords:** Companion animals, Parasites, Economic impact, Veterinary expenses, Parasiticides

## Abstract

**Background:**

Dogs and cats in the UK are routinely exposed to a range of internal and external parasites that can adversely affect their health and welfare. Among endoparasites, roundworms and lungworms are of particular concern, while ectoparasites such as fleas and ticks remain persistent veterinary challenges. Although preventive parasiticide use is widespread, its broader health and economic benefits have not been comprehensively quantified. Moreover, evolving parasite epidemiology and increasing scrutiny of the environmental impacts of some treatments have prompted debate about their continued use. This study presents a preliminary modelling framework designed to estimate the health and economic benefits of parasite prevention in UK companion animals, focusing on averted infections and associated veterinary cost savings.

**Methods:**

A spreadsheet-based model was developed using available data on parasite prevalence, parasiticide usage, treatment frequency, and estimated veterinary costs, supplemented by expert consultation. Two scenarios were assessed: (A) current parasiticide coverage, and (B) optimal coverage assuming full adherence to recommended treatment guidelines.

**Results:**

Preliminary findings suggest that existing parasite control measures targeting *Toxocara canis*, *Toxocara cati*, *Angiostrongylus vasorum*, fleas, and ticks prevent approximately 5.5 million infections in UK dogs and cats each year. Under optimal compliance, the number of averted infections could increase by 70.6%, reaching 9.3 million annually. Economically, current prevention practices are estimated to save UK households around £53 million per year in veterinary costs, with potential savings rising to £95.2 million under optimal coverage and reduced parasite prevalence.

**Conclusions:**

These findings underscore the substantial health and economic value of preventive parasite control in UK pets. While the model relies on simplified assumptions and available data that may overrepresent higher-risk populations, it provides a valuable foundation for future refinement. Incorporating more granular epidemiological, geographical, and seasonal data will enhance precision. As a preliminary framework, this model reinforces the importance of preventive strategies and highlights the potential for even enhanced impact through improved compliance and targeted interventions.

**Graphical Abstract:**

**Figure:**
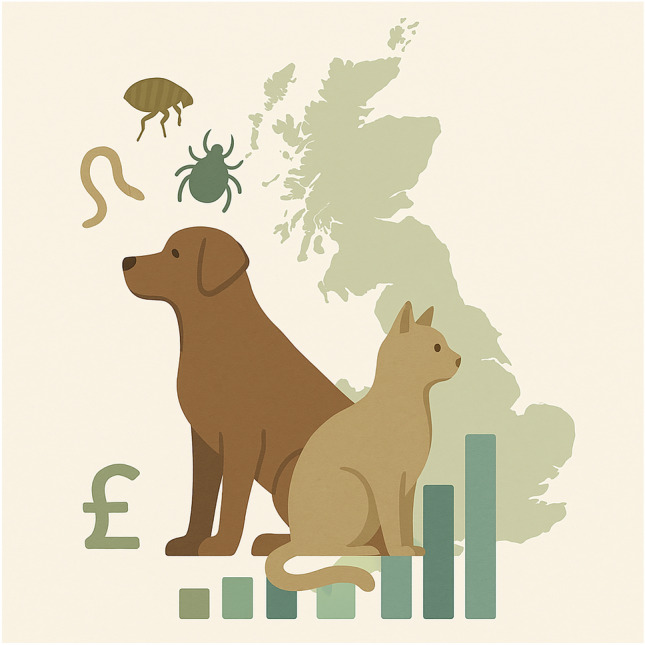

## Background

Parasitic infections in dogs and cats occur worldwide and represent an important One Health concern owing to the close and frequent contact between pets and humans [1–3]. The strong emotional bond owners share with their pets, often regarded as family members, can inadvertently increase human exposure to zoonotic parasites [4–6]. In many countries, parasite prevention in companion animals relies on well-established practices, including parasiticide administration and diagnostic testing [7–9].

However, growing concerns have emerged about the development of antiparasitic resistance in companion animal parasites [10]. Reports from the USA have confirmed widespread benzimidazole resistance in *Ancylostoma caninum* [11], while studies from Australia documented pyrantel resistance [12]. Although resistance in companion animals has not yet reached the critical levels seen in livestock parasites [13], the threat is significant; particularly for zoonotic parasites such as *Toxocara* spp. [14] and *Echinococcus* spp. [15]. These concerns underscore the need for strategic, evidence-based anthelmintic use within a holistic One Health framework [10].

Over recent decades, shifts in global pet ownership, increased animal movement, and widespread prophylactic treatments have reshaped parasite epidemiology. These changes have reignited debate over the relative merits of regular versus targeted parasite control in pets [16, 17]. While the importance of prevention is widely acknowledged, its quantitative health and economic benefits remain poorly defined in veterinary medicine. Human health research has similarly focused on a narrow subset of high-impact zoonotic parasites, leaving broader implications underexplored [18].

At the same time, the preventive use of parasiticides is being increasingly questioned; particularly regarding their environmental footprint and unintended effects on nontarget organisms [19]. A lack of robust, quantitative data on the health and societal benefits of parasite prevention hampers informed discussion of these risk–benefit trade-offs. Encouragingly, recent developments, such as the first global burden of disease estimates for toxocariasis [20], illustrate a growing drive to establish meaningful, cross-sector metrics to guide One Health decision-making.

Despite this progress, parasite surveillance in companion animals remains limited. Key challenges include the separation of human and veterinary health data, the absence of integrated information systems, and the underuse of quantitative methods linking pet parasite data to public health outcomes [18]. In the UK, surveillance has largely been advanced through research initiatives such as the Small Animal Veterinary Surveillance Network (SAVSNET) and VetCompass [21–25]. SAVSNET captures real-time clinical and laboratory data to monitor disease trends and antimicrobial use, while VetCompass analyzes anonymized practice records to investigate disorder prevalence, risk factors, and outcomes. Together, these initiatives inform veterinary public health, research, and policy; but crucial health and economic indicators for the wider pet population remain undefined.

This study seeks to help fill that gap by developing a quantitative framework to assess the impact of parasites and their control measures in UK dogs and cats. Focusing on four key parasite groups, *Toxocara canis* and *Toxocara cati* (ascarids), *Angiostrongylus vasorum* (lungworm), fleas, and ticks, we evaluate associated health risks in both protected and unprotected pets. Using the best available data, we provide preliminary prevalence estimates, assess the likely veterinary and economic costs, and highlight opportunities for more evidence-based, One Health-aligned parasite control strategies in companion animals.

## Methods

### Model overview

To assess the potential impact of current and optimal parasite control strategies in companion animals, we developed a quantitative model focusing on pet health outcomes and pet owner expenses. The framework was applied to the UK, where relatively robust data exist on parasite prevalence and parasiticide usage.

### Impact on pet health and owner costs

**Fig. 1:**
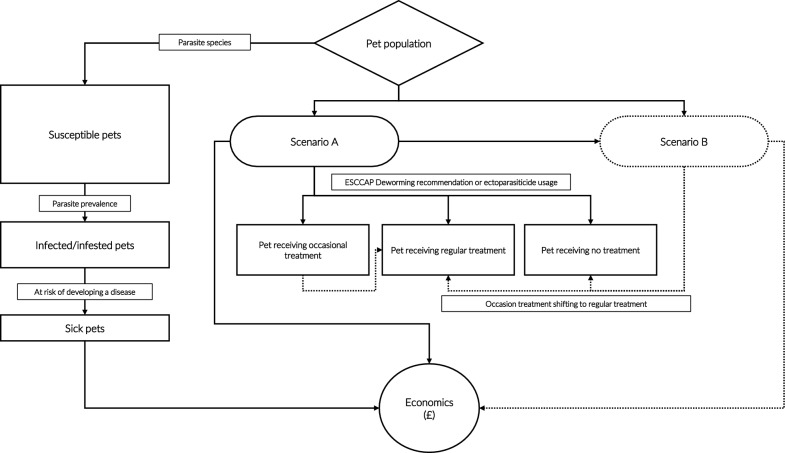
Diagram depicting the calculation approach, with relationship between parasite species, pet susceptibility, and treatment scenarios

The model comprised multiple compartments and fixed variables (Table 1), categorizing the pet population (dogs and cats) into three groups: susceptible, infected/infested, and clinically affected (Fig. 1). Infection (endoparasites) or infestation (ectoparasites) prevalence was derived primarily from national literature sources; where data were lacking, expert opinion was used (Table 1). When available, uncertainty intervals (UI) were included on the basis of primary sources.

**Table 1:** Model input parameters used for estimating pet health outcomes and economic impacts, with associated data sources

Parameter		
Pet population:	Number of individuals	References
Dogs	12,000,000	[82]
Cats	11,000,000	[30]
Parasite prevalence (pet):	Prevalence (95% CI)	References
*Toxocara canis* (dog)	4.4% (1.6–8.5%)	[57]
*Toxocara cati* (cat)	18.8% (9.4–28.1%)	[58]
*Angiostrongylus vasorum* (dog)	1.3% (1.0–1.7%)	[83]
Fleas (dog)	14.4% (11.7–17.1%)	[69]
Fleas (cat)	28.1% (25.0–31.2%)	[69]
Ticks (dog)	30.7% (29.6–31.8%)	[75]
Ticks (cat)	6.6% (4.7–8.5%)	[84]
Clinical alterations:	Prevalence	References
Diarrhea due to *Toxocara* spp. in young pets	1.4%	[61]
Clinical canine angiostrongylosis among infected dogs	15.7%	[85]
Flea allergic dermatitis (in infested dog)	0.48%	[41]
Flea allergic dermatitis (in infested cat)	1.5%	[42]
Dogs referred to vets for flea infestation	2.05%	[41]
Cats referred to vets for flea infestation	5.01%	[42]
Dogs referred to vets for tick infestation	0.68%	[41]
Treatment adherence, Scenario A:	Parameter and value	References
Treatment for endoparasites*	Year-round compliance: Dogs: 8.6–25% Cats: 11.8–37.1% Frequency of treatment/year: Dogs: 2–3.5 times Cats: 2.4–3.4 times	Estimated from [26, 27]
Treatment for ectoparasites*	Animals under regular treatment: Dogs: 36.1% Cats: 31.7% Average protection of 3 doses of parasiticide: Fleas: 4.5–5.1 months Ticks: 4.8–5.1 months	Estimated from [19, 29]
Treatment adherence, Scenario B:	Proportion (95% UI)	References
Scenario B, deworming (dog)	85.7% (83.5–87.9%)	Estimated from [86]
Scenario B, deworming (cat)	77.7% (76.1–79.3%)	Estimated from [86]
Scenario B, treatment of fleas (dog)	78.2% (75.8–80.6%)	Estimated from [86]
Scenario B, treatment of fleas (cat)	82.0% (80.3–83.7%)	Estimated from [86]
Scenario B, treatment of ticks (dog)*	80%	Estimated from [86]
Scenario B, treatment of ticks (cat)*	50%*	Estimated from [86]
Scenario B, prevention of *A. vasorum*	44%	[85]
Economics, cost of treatment	Estimated costs	References
Clinical canine angiostrongylosis	£ 1500	[87]
Treatment of flea allergic dermatitis	£ 300	[88]
Treatment of diarrhea during toxocariosis in young animals	£ 250	[89]
Flea treatment at home using can spray	£ 20	[90]
Veterinary examination and flea or tick treatment	£ 60	[41]

*UI = uncertainty interval. Values marked with an asterisk (*) lack published uncertainty estimates and should therefore be considered approximate

The number of treated and untreated animals was estimated using published data on parasiticide usage, distinguishing between regular, occasional, and no treatment categories (Table 1, *Treatment adherence*). Given the absence of official compliance data, two hypothetical scenarios were modeled. Scenario A, reflecting current likely treatment practices in the UK, and Scenario B, representing optimal coverage, with full adherence to treatment guidelines. Direct health costs were estimated from the probability that infected or infested animals would develop clinical disease requiring veterinary examination, diagnostics, and treatment (Table 1).

### Infection status

Animals were considered susceptible based on the reported prevalence of the target parasites. Pets testing positive for a parasite were categorized as infected or infested, and a proportion of these were assumed to develop observable clinical disease, as reported in reference data. Estimates of clinical prevalence were then used to calculate the number of affected animals within the total population (Table 1).

### Protection status and modelling scenarios

The impact of parasiticide treatment on the number of prevented cases and associated cost savings was assessed under two modeled scenarios (Table 1). This approach was necessary due to limited large-scale, reliable data on compliance in companion animal parasite control. Scenario A represented current coverage, in which pets were classified into three groups: regularly treated, occasionally treated, or untreated. These estimates reflect current practices in the UK and were informed by national survey data on deworming recommendations [26] and ectoparasiticide usage [19]. Scenario B represented optimized coverage, assuming that all pets receiving occasional treatment under Scenario A would instead receive regular, guideline-aligned treatment. Estimates for this scenario were derived from annual PDSA reports documenting deworming and flea treatment behaviors among UK pet owners (Table 1). To account for differences in administration frequency and duration of efficacy, treatments for endoparasites and ectoparasites were modeled separately.

### Treatment against endoparasites

For Scenario A, pets were categorized according to regular versus occasional deworming, following European Scientific Council for Companion Animal Parasites (ESCCAP) guidelines [27]. Classification was based on survey data linking parasite risk levels and deworming frequency among UK pet owners [26]. ESCCAP-defined risk classes were aligned with reported treatment frequencies to estimate the number of dogs and cats receiving regular or occasional deworming nationally (Table 1, *Treatment compliance*).

Adherence to guidelines was quantified by dividing the reported mean annual number of deworming treatments by the number of months during which ESCCAP recommends treatment for each risk category [27] (Tables 2 and 3). Pets that received no deworming were considered untreated.

**Table 2:** Estimated adherence to ESCCAP-recommended deworming frequencies by risk group and animal species

Species	Risk group*	ESCCAP recommended deworming frequency	Frequency adopted in the model	Alignment to estimated frequencies
Dogs	A	1–2 times per year	2 times per year	N/A**
	B	4 times per year	4 times per year	2.0 / 4 (50.0%)
	C	> 4 times per year	8 times per year	3.5 / 8 (43.8%)
	D	Once a month	12 times per year	3.2 / 12 (26.7%)
Cats	A	1–2 times per year	2 times per year	N/A**
	B	At least 4 times per year	4 times per year	2.4 / 4 (60.0%)
	C	> 4 times per year	8 times per year	2.9 / 8 (36.3%)
	D	Once a month	12 times per year	3.4 / 12 (28.3%)

*Risk groups (A–D) are defined according to ESCCAP guidelines based on lifestyle and exposure risk. Dogs: Group A: dogs > 6 months old, mostly indoor or going outside without direct contact with parks, other animal feces, snails/slugs, prey, or raw meat. Group B: dogs > 6 months old, go outside and have contact with parks or other animal feces, but do not eat prey, snails/slugs, or raw meat. Group C: dogs > 6 months old, go outside, have contact with parks or other animal feces, and eat prey, snails/slugs, or raw meat. Group D: dogs < 6 months old, or living in areas endemic for *Echinococcus multilocularis*, or hunt/eat prey, or live indoors, eat raw meat, and live with children/elderly. Cats: Group A: indoor cats, low exposure to worm stages, unlikely to eat rodents. Group B: outdoor cats, high exposure to worm stages, likely to eat rodents. Group C: cats that eat prey and/or hunt outdoors, and consume raw meat. Group D: outdoor cats living with young children or immunocompromised individuals

**N/A indicates that comparison was not applicable because no animals are reported within this risk category A

**Table 3:** Average annual duration of protection against ectoparasites in dogs and cats based on three treatment units per year

Parasite group	Cumulated months of protection in dogs/year *	Cumulated months of protection in cats *
Fleas	Up to 4.5	Up to 5.1
Ticks	Up to 4.8	Up to 4.9

*Values derived from the UK Veterinary Medicines Directorate product information database [29]

### Treatment against ectoparasites

The proportion of dogs and cats receiving regular, year-round ectoparasiticide treatment was estimated from UK data on companion animal ectoparasiticide usage [19] (Table 1, *Treatment compliance*). As ESCCAP risk categories are not defined for ectoparasites, compliance was assumed to vary according to perceived parasite risk. For fleas, treatment was considered regular when administered continuously throughout the year. For ticks, regular treatment was defined as at least 8 months of protection annually, reflecting the typical UK tick season (March–October) [28], though year-round exposure was also acknowledged in milder climates.

Pets receiving occasional treatment were defined as those whose owners purchased a single package of ectoparasiticide per year. On the basis of an analysis of the UK Veterinary Medicines Directorate (VMD) Product Information Database [29], a typical package was assumed to contain an average of three treatment units. Occasional users were thus considered unlikely to repurchase or complete a full course. The average protection duration per treatment unit was derived from VMD data (Table 3). Using these assumptions, the proportions of regularly, occasionally, and untreated pets were estimated, enabling calculation of the number of cases prevented and the associated economic benefits.

### Scenario B: increasing compliance with treatment

Scenario B modeled the effect of improving compliance by converting all occasionally treated animals to regular treatment. Pets were classified as “treated” if they received any preventive parasiticide, endoparasiticide, ectoparasiticide, or endectocide, at any point during the year. Estimates were based on PDSA report data from the past decade, describing owner-reported flea prevention and deworming behaviors (Table 1, *Treatment compliance*). Where specific data were unavailable (e.g., for tick or *A. vasorum* treatment), rates were inferred from PDSA reports or approximated using companion animal parasiticide market values. Pets receiving no treatment during the year were considered untreated.

### Cost of treatment in clinically affected animals

The total cost of treating clinically affected animals was estimated from average veterinary expenses incurred by pet owners, including examination, diagnostics, hospitalization, and therapy (Table 1, *Economics, cost of treatment*). Cost data were obtained from informal surveys of three veterinary clinics located in Edinburgh, Birmingham, and London. These values represent the average cost to pet owners if preventive treatment had not been applied. For fleas, additional household treatment costs were included, as infestations typically require environmental decontamination. These costs were estimated by combining data on the number of UK households with dogs and cats (8.7 million and 7.3 million, respectively [30]), the prevalence of flea infestation (Table 1, *Parasite prevalence*), and the average retail price of household flea sprays.

## Results

### Impact of regular or occasional treatment on pet population metrics

**Fig. 2:**
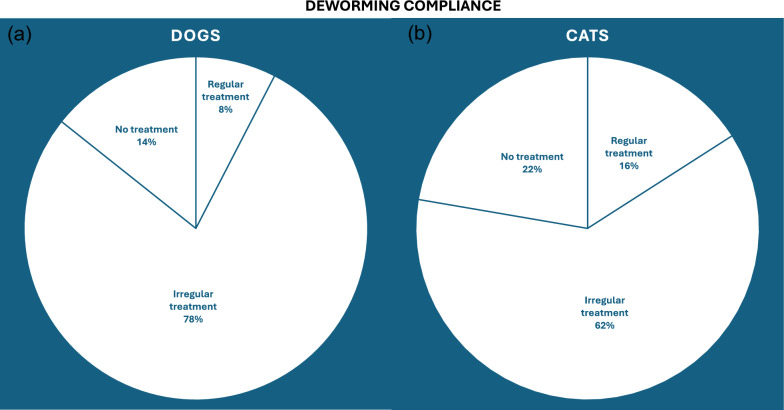
Deworming adherence rates with percentages of irregular, regular, and no treatment in dogs (**a**) and cats (**b**)

Under Scenario A, modelling based on ESCCAP deworming guideline compliance indicated that 7.2% of dogs (approximately 860,000) receive regular monthly endoparasite treatment throughout the year, while 73.8% (around 8.9 million) are treated only occasionally (Fig. 2a). Across risk groups B, C, and D [ESCCAP, 2021], approximately 145,800, 97,200, and 8.6 million dogs, respectively, receive an average of 2.0, 3.5, and 3.2 annual doses of dewormer (Table 2). These correspond to 50.0%, 43.8%, and 26.7% of ESCCAP’s recommended dosing frequency. Among cats, 14.6% (1.6 million) receive regular monthly deworming, while 56.4% (3.2 million) are treated occasionally (Fig. 2b). Annually, approximately 1.4 million, 207,000, and 4.6 million cats in risk groups B, C, and D, respectively, receive an average of 2.4, 2.9, and 3.4 doses—equating to 60.0%, 36.3%, and 28.3% of ESCCAP recommendations (Table 2).

**Fig. 3:**
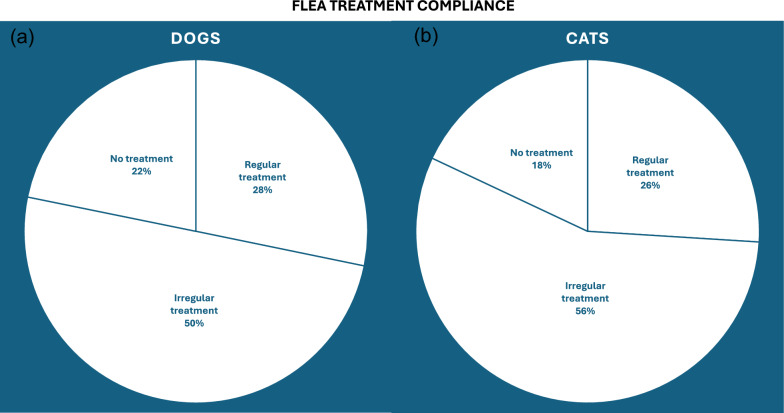
Comparison of ectoparasiticide treatment adherence for fleas (**a**) and ticks (**b**) in dogs

**Fig. 4:**
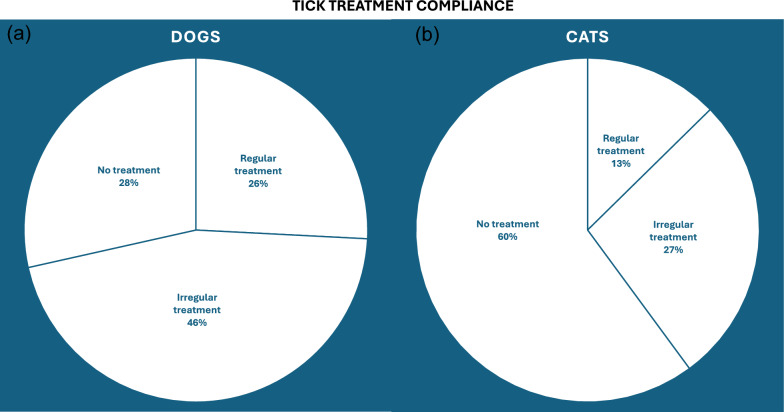
Comparison of ectoparasiticide treatment adherence for fleas (**a**) and ticks (**b**) in cats

For ectoparasite control, 28.9% of dogs (up to 3.5 million) and 19.0% of cats (around 2.1 million) receive regular year-round flea treatments, while an additional 51.2% of dogs and 41.0% of cats are treated occasionally (Figs. 3a and 4a). Assuming each owner purchases a pack containing three doses, approximately 6.1 million dogs and 4.5 million cats receive only 38% and 43%, respectively, of the doses required for continuous protection. For tick control, 25.8% of dogs (around 3.1 million) and 12.6% of cats (1.4 million) receive regular seasonal treatment, whereas 45.7% of dogs and 27.2% of cats receive occasional treatment (Figs. 3b and 4b). Consequently, 5.5 million dogs and 3.0 million cats achieve approximately 60% and 64% of the recommended dosing coverage for the UK’s 8-month tick season.

Under Scenario B, representing full adherence to recommended preventive schedules, 10.3 million dogs and 8.5 million cats would receive regular annual deworming, leaving 1.7 million dogs and 2.5 million cats untreated. For ectoparasites, 9.4 million dogs would receive flea prevention and 9.6 million tick prevention, while 9.0 million cats and 5.5 million would receive treatment for fleas and ticks, respectively. Transitioning from occasional to regular treatment would lead to a 196.7% and 104.1% increase in prevented roundworm cases in dogs and cats, respectively. For ectoparasites, the number of prevented cases would rise by 66.5% (fleas) and 50.3% (ticks) in dogs, and 64.7% (fleas) and 90.3% (ticks) in cats. Overall, this would generate an estimated 66% increase in household savings on flea-related costs.

### Impact on overall pet health and owner costs

Under Scenario A, current parasite control measures in the UK are estimated to prevent up to 5.5 million infections or infestations annually (95% UI 4.5–6.4 million), resulting in total household savings of £53 million (95% UI £45.3–61.3 million). Of this, £20.7 million (95% UI £17.4–24.5 million) are direct veterinary healthcare costs (consultation through treatment), and £32.2 million (95% UI £27.9–36.8 million) are flea-related home treatment expenses. If pets receiving occasional or irregular treatment were to adopt consistent, regular control (Scenario B), the number of prevented cases could increase by 70.6%, reaching 9.3 million annually (95% UI 9.2–9.4 million). Corresponding household savings would increase by 79.7%, reaching £95.2 million per year (95% UI £93.3–96.8 million), comprising £41.7 million (95% UI £41.1–42.0 million) in veterinary expenses and £53.5 million (95% UI £52.2–54.8 million) in home flea control costs.

### Impact of *Toxocara canis* and *Toxocara cati* control

Current parasiticide use targeting roundworms is estimated to prevent approximately 152,500 cases of *T. canis* in dogs and 787,200 cases of *T. cati* in cats each year (Scenario A, Table 4). This equates to approximately 210 cases of gastrointestinal illness in puppies and 2425 in kittens. These preventive measures yield estimated savings of £51,780 for dog owners and £606,140 for cat owners annually. Under Scenario B, regular deworming would prevent approximately 452,500 *T. canis* cases and 1.61 million *T. cati* cases each year, generating an estimated £153,620 and £1.24 million in veterinary savings for dog and cat owners, respectively.

**Table 4:** Estimated number of parasite cases prevented and associated household savings in the UK under current treatment practices (Scenario A)

Estimated health and economic impact	*T. canis*	*T. cati*	*A. vasorum*	Fleas		Ticks	
	Dogs	Cats	Dogs	Dogs	Cats	Dogs	Cats
Prevented cases (*n*)							
Cases prevented (regular protection)	40,010	329,353	6069	487,818	803,475	950,896	91,827
Cases prevented (occasional protection)	112,489	457,843	17,064	323,804	735,737	1,009,899	98,924
Total cases prevented	152,499	787,196	23,133	811,622	1,539,211	1,960,794	190,750
Household savings (£)							
Household savings (regular protection)	13,583	253,601	1,456,560	1,302,474	6,030,880	387,967	N/A
Household savings (occasional protection)	38,190	352,538	4,095,360	864,557	5,522,440	412,037	N/A
Total savings (£)	51,773	606,140	5,551,920	2,167,031	11,553,320	800,004	N/A*

*N/A = no reliable economic data available for this species. Values represent modeled estimates under Scenario A; totals may not sum precisely due to rounding

### Impact of *A. vasorum* control

Preventive treatments effective against *A. vasorum* are estimated to avert 23,100 canine infections annually under Scenario A (Table 4), preventing approximately 3700 symptomatic cases of canine angiostrongylosis. Transitioning to regular preventive treatment (Scenario B) would prevent an estimated 68,640 infections annually, a nearly 200% increase, and generate household savings of approximately £16.47 million in veterinary expenses across the UK.

### Impact of flea control

Parasiticides effective against fleas are estimated to prevent 811,600 flea infestations in dogs and 1.5 million in cats each year (Scenario A, Table 4). This likely prevents approximately 16,600 veterinary consultations for dogs and 77,100 for cats, as well as 3900 cases of flea allergy dermatitis (FAD) in dogs and 23,100 in cats. Flea prevention is estimated to save £2.2 million (dogs) and £11.6 million (cats) in veterinary costs annually. Without preventive control, around 3.3 million UK households would need to implement additional pest management—such as insecticidal sprays—at an estimated cost of £66.4 million (£25.2 million among dog owners and £41.2 million among cat owners). Consequently, flea prevention is estimated to save households £32.2 million annually (£11.8 million for dog owners, £20.4 million for cat owners) under Scenario A. Under Scenario B, an estimated 1.4 million dog and 2.5 million cat flea cases could be prevented each year, generating an additional £3.6 million (dogs) and £19.0 million (cats) in veterinary cost savings, and up to £53.5 million in total household pest control savings.

### Impact of tick control

Tick prevention measures are estimated to avert 1.9 million infestations in dogs and 190,700 in cats each year under Scenario A (Table 4). This reduction corresponds to approximately 13,330 fewer veterinary consultations for dogs, resulting in an estimated annual household saving of £800,000. Comparable data for cats were unavailable. Under Scenario B, representing full adherence to preventive guidelines, regular treatment could prevent an estimated 2.9 million tick infestations in dogs and 363,000 in cats each year, yielding projected veterinary cost savings of approximately £1.2 million annually for UK households.

## Discussion

This study provides preliminary quantitative evidence that optimizing parasite control in companion animals can deliver substantial health and economic benefits. The results emphasize the need for enhanced surveillance, stronger adherence to evidence-based guidelines, and implementation of risk-targeted preventive strategies to protect both animal and public health.

This study sought to quantify the combined animal health, social, and economic impacts of parasite prevention in UK dogs and cats. Effective parasite control benefits both animal welfare and human health, yet quantitative studies comparing prevention strategies remain scarce [31]. Surveillance of zoonotic diseases associated with companion animals is inconsistent across countries, ranging from mandatory reporting to voluntary data sharing [18]. For example, the World Organisation for Animal Health’s WAHIS database primarily monitors zoonoses affecting livestock and wildlife (e.g., avian influenza, African swine fever, coronavirus disease 2019 (COVID-19)), with limited representation of companion animal diseases. Visceral leishmaniasis is one of the few exceptions, although most research on this disease focuses on human health rather than animal welfare [32–34].

Quantitative assessments of endo- and ectoparasites in dogs and cats are typically confined to descriptive epidemiological studies reporting prevalence, distribution, and risk factors [18]. In contrast to human and livestock diseases, companion animal parasitic diseases are rarely included in coordinated international surveillance systems; exceptions being rabies, and to some extent leishmaniasis and echinococcosis in humans [35–38]. This highlights an urgent need for robust, standardized monitoring frameworks that assess the health and economic impacts of parasitic infections in both animals and people.

Data-driven initiatives, such as SAVSNET [39] and VetCompass in the UK and Australia [40], are helping to bridge this gap. SAVSNET demonstrates the potential of electronic veterinary health records to track disease trends, while VetCompass provides large-scale prevalence data on common disorders in dogs and cats [41, 42]. Together, these platforms facilitate evidence-based decision-making by veterinarians, public health authorities, and pet owners, improving understanding of disease dynamics and enabling proactive interventions.

Our analysis confirms that the risk of parasitic infection in companion animals varies by species and is strongly influenced by owner compliance with parasiticide use. While high parasite prevalence increases infection risk [6, 43], compliance is a reciprocal driver; it both shapes and responds to prevalence trends. Regular and correctly timed administration of parasiticides is therefore essential for maintaining protection. Tailoring preventive strategies to local epidemiological conditions can maximize benefits and reduce the broader health and economic burden on households and society [44–46].

In Scenario B, representing full compliance with established protocols, the model estimated a 78.4% increase in the number of parasite cases prevented, accompanied by a significant reduction in prevalence. Despite the existence of clear, evidence-based guidelines, inconsistent owner adherence remains a major barrier. Surveys from Finland [47], Australia [48], Ireland [49], France [50], Spain [51], Portugal [52], Germany [53], and Italy [54] demonstrate wide variability in compliance, even in regions with strong veterinary infrastructure. Such inconsistency limits the effectiveness of parasite prevention and perpetuates zoonotic risks [26].

Our findings suggest that animals treated regularly achieve sustained reductions in infection risk, while the effectiveness of occasional treatments depends heavily on timing relative to exposure; typically within 1 month for endoparasiticides and 1–8 months for ectoparasiticides [46]. This study used ESCCAP guidelines as the benchmark due to their scientific rigor, species-specific recommendations, and broad European adoption. We acknowledge, however, that organizations such as the BVA, BSAVA, and BVZS advocate for more targeted and environmentally sustainable approaches [55]. Our model does not prescribe a single standard; rather, it explores the consequences of improving compliance under a widely recognized framework. Ultimately, optimal parasite control should be risk-based, informed by regional epidemiology, guided by diagnostic testing, and supported by veterinary expertise [11, 56].

The results reaffirm the importance of *T. canis* and *T. cati* as major roundworms in UK dogs and cats, underscoring the compliance challenges in controlling these parasites [57, 58]. Although often associated with juveniles, where signs such as a “pot-bellied” appearance are typical, *Toxocara* infections can also occur in adults, causing gastrointestinal disturbances and, in severe cases, requiring hospitalization [59–61]. Toxocariasis remains a neglected zoonosis [62] with an estimated global economic burden of $2.5 billion and approximately 91,700 DALYs lost annually [20, 63].

Among lungworms, *A. vasorum* represents the most clinically significant species in UK dogs [64]. Its distribution has expanded from southern England to Scotland, with regional variations influenced by fox populations as reservoir hosts [65, 66]. Clinical manifestations range from asymptomatic infection to severe multisystemic disease affecting the nervous, cardiovascular, coagulation, and gastrointestinal systems [67].

Fleas continue to be the most prevalent ectoparasites in UK companion animals. *Ctenocephalides felis* and *Ctenocephalides canis* dominate owing to their adaptability and year-round persistence in domestic environments [68–71]. In addition to causing irritation and flea allergic dermatitis [72], fleas can transmit *Dipylidium caninum* and *Bartonella* spp. [73]. Because flea infestations frequently lead to pest control interventions, monitoring pest management service data could enhance understanding of the societal costs associated with these infestations.

Ticks remain globally significant vectors of viral, bacterial, and protozoan pathogens [6]. In the UK, the distribution of *Ixodes ricinus*—the primary vector of tick-borne encephalitis and Lyme borreliosis—has expanded substantially in recent decades [74]. Other species such as *Ixodes hexagonus*, *Ixodes canisuga* [75], and *Dermacentor reticulatus* (vector of *Babesia canis*) are increasingly reported [76]. Key drivers include climate change, movement of untreated pets, habitat modification and shifting wildlife populations [77]. Despite growing knowledge of canine tick-borne diseases, significant gaps remain in feline surveillance, limiting the development of effective control strategies [78].

Beyond the parasites analyzed here, others also impose societal costs. Canine tapeworms, such as *Taenia ovis* and *Taenia hydatigena*, cause over £11 million in annual UK losses through carcass and offal condemnation [79]. Dogs may also act as definitive hosts for *Echinococcus granulosus*, a zoonotic tapeworm whose distribution appears broader than previously thought, with coproantigen prevalence of up to 10.6% in farm dogs and 1.63% in strays [80]. Although prevalence in pets is likely lower, their potential reservoir role warrants attention. Furthermore, *D. caninum*, while typically of minor clinical concern, may show emerging antiparasitic resistance and remains relevant to discussions on sustainable parasite management [81].

Our study has several limitations. First, the analysis drew on variables from published literature and publicly available datasets, many of which lack recent updates or sufficient granularity. Comprehensive prevalence data, including spatial risk maps, longitudinal infection dynamics, co-infection patterns, and comparative data from treated versus untreated animals in natural settings, were not available at the time of modelling. Similarly, data describing socio-economic drivers of owner behavior (e.g., treatment affordability, awareness, or access to veterinary care) remain limited. Second, the model focused on a subset of parasites and clinical conditions, excluding diseases such as giardiasis, toxoplasmosis, and vector-borne infections owing to insufficient UK-wide prevalence data. Other species, including hookworms and *Aelurostrongylus abstrusus*, likely contribute to the overall parasitic burden and warrant inclusion in future analyses. Human health implications, such as zoonotic *Toxocara* infection and flea- or tick-borne diseases, were also beyond this model’s scope. In addition, the model assumes that effective parasiticide treatment leads to proportional reductions in prevalence and does not account for incomplete dosing, improper administration, potential drug resistance, or reinfection. While these simplifications allow for broad inference, they limit the model’s ability to fully represent complex epidemiological dynamics.

## Conclusions

This study provides quantitative evidence that current preventive parasite treatments in UK dogs and cats deliver substantial health and economic benefits. These benefits extend beyond individual animal welfare, influencing public health, veterinary economics, and the human–animal bond. The findings highlight the need for enhanced surveillance, enhanced owner compliance, and risk-based treatment strategies. At the same time, it is important to acknowledge growing evidence of the environmental consequences of widespread antiparasitic use, including contamination of aquatic systems and harm to non-target species. A balanced One Health approach—integrating animal, human, and environmental perspectives—is essential for developing sustainable, evidence-driven parasite control policies in the future.

## Data Availability

The data supporting the findings of the study are available within the article.

## Abbreviations

BSAVA: British Small Animal Veterinary Association
BVA: British Veterinary Association
BVZS: British Veterinary Zoological Society
DALYs: Disability-adjusted life years
ESCCAP: European Scientific Council for Companion Animal Parasites
PDSA: People’s Dispensary for Sick Animals
SAVSNET: Small Animal Veterinary Surveillance Network
UI: Uncertainty intervals
UK: United Kingdom
VMD: Veterinary Medicines Directorate
WAHIS: World Animal Health Information System
